# Stigmatization, Discrimination, Racism, Injustice, and Inequalities in the COVID-19 Era

**DOI:** 10.34172/ijhpm.2020.87

**Published:** 2020-06-03

**Authors:** Jaime A. Teixeira da Silva

**Affiliations:** Independent Researcher.

## Dear Editor,

The pandemic caused by the severe acute respiratory syndrome coronavirus 2 (SARS-CoV-2), which induces the respiratory coronavirus disease 2019 (COVID-19) and has transformed society, medicine, and values in so many ways, has already claimed (May 31, 2020) 369 627 lives and infected 6 087 902 people globally.^1^ During this highly transformative period, human interactions have been forcefully altered, either through implemented social distancing or lockdowns, to limit the spread of the virus. Reducing people’s movement, and the duration and frequency of contact between them, imposes artificial barriers of contact that can lead to friction and discord. Healthcare systems, often under pressure, were unprepared and faced life-and-death decisions, reigniting or amplifying sensitive social issues, including stigmatization, discrimination, racism, injustice, and inequalities, as a result of health disparities.^2^

In this time of isolation, affected individuals have largely stayed in contact through social media and online apps, but the use of social media has also seen a rise in misinformation^3^ and fake news,^4^ which may negatively impact the health and lives of individuals, especially those seeking advice regarding sanitary conditions or treatments to halt or prevent COVID-19. Compounding the immediacy of the pandemic, patients infected by this virus may be receiving prioritized treatment at the expense of patients with other critical illnesses.^5^ The strain on human resources, particularly healthcare workers, is acute when essential care and equipment becomes scarce, leading to competition between critical care and emergency cases, or even negligence of the elderly, those with special disabilities, as well as indigenous, homeless, migrant and imprisoned populations.^6^ Border closure, limiting the international or transnational movement of individuals, or imposed travel restrictions, worsened the plight of asylum seekers and undocumented migrants.^7^ These travel restrictions might be a violation of International Health Regulations if the restriction infringes upon rights of movement to seek a better or viable health solution.^8^

There was a rise in anti-Chinese sentiment, or racism, causing shame and stress to Chinese nationals or even to other Asians, and thus stigmatization.^9^ Such prejudice often arises with the need for self-protection, and the fear underlying this need for a racialized response to the COVID-19 threat led to some panic and hysteria, microaggressions and mass generalizations, even spates of violence and protests, causing the disproportional victimization of ethnic minorities and socio-economic discrimination of marginalized groups, made worse by erroneous misinformation.^10^

Curtailed movement and choices, or intrusive and forcefully imposed policies, restrictions or regulations, the use of state surveillance, drones, forced implementation of mobile apps to track citizens and the spread of COVID-19, color coding for citizens based on their travel and health status, as well as profiling, even under the premise of protecting society’s health, may be used to fortify state control and surveillance, possibly restricting civil liberties, or freedoms and rights, thereby raising privacy concerns by suspending democratic deliberation or fortifying authoritarianism.^11,12^ COVID-19 might fortify other psychosocial and structural burdens, including misogyny, homophobia, homelessness and mental health.^13^

In this pandemic, and in post-COVID-19 societies, the challenges that humanity faces as a result of limitations need to be addressed now, prior to the arrival of the next pandemic, or the resurgence of this one. If the healthy versus sick dividing line can be better appreciated through solid science- and evidence-based medicine, and if proper public health governance and policies can be effectively implemented through resolute political leadership, then more compassion towards those infected by COVID-19, or respect towards others that are mistreated as a result of this pandemic, including those who recovered from COVID-19 and are living a post-infection life, may emerge and prevail.^14,15^

## Ethical issues

Not applicable.

## Competing interests

Author declares that he has no competing interests.

## Author’s contribution

JATdS is the single author of the paper.
